# Mr. Bradley's Cases in Surgery, &c.

**Published:** 1801-04

**Authors:** 


### Mr. BRADLEY's *Cases in Surgery, &c.*

[ Continued from our laſt, pp. 219—223. ]

### *Case of Triſmus, or Locked-Jaw.*

LYDIA KAYE, aged 20 years, of a full habit and ſanguine conſtitution, and fifteen weeks advanced in her pregnancy, had the misfortune to receive a blow from the head of a floor-bruſh, on the back part of her head, at its articulation, which knocked her down. She was taken up ſenſeleſs, and laid on a bed. Three hours afterwards, I found her in the ſame poſition in which ſhe was laid. Her head was thrown backwards, and inclined rather to the right ſide. The part on which the blow was inflicted, was ſomewhat full and painful, but without any diſcolouration or muſcular rigidity. Her pulſe was quick and fluttering, and her reſpiration panting, and very frequent. Her jaw was locked ſo cloſe, as to render it very difficult to paſs the handle of a table ſpoon flat-wiſe betwixt her teeth. On pouring a little water from a tea ſpoon into her mouth, ſhe ſeemed to make one effort to ſwallow; but, on repeating it ſeveral times, no further attempt at ſwallowing was made, and the water regurgitated, without exciting any apparent action of the ſtomach or lungs. The eye-lids were about two-fifths open, and were looſe, and afforded no reſiſtance on opening them: The upper, when elevated, might be about ten or twelve ſeconds in receding to their former poſition. The eye-balls were drawn upwards, and fixed, inſomuch, that little more than the verge of the cornea was diſcernible; the pupil alſo was very much dilated, and exhibited not the leaſt contraction on being expoſed to the light. She was hot, and perſpired freely, eſpecially on the cheſt. No further injury ſeemed to have been done to any other part of the body, except a ſlight contuſion on the left temple, extending downwards to the articulation of the lower jaw. This part, on preſſure, always afforded her pain, and which was known by her moaning and moving her right arm, although theſe two ſymptoms ſometimes exiſted independently of this cauſe. The maſſeter-muſcles were full, and contracted, eſpecially the left.

On attributing theſe ſymptoms in ſome meaſure to *hyſteria*, a ſolution of *emetic tartar* was ordered to be taken at intervals; and notwithſtanding her inability to ſwallow, a quantity was taken in the courſe of the night, ſufficient to puke her twice, but without affording her any kind of relief: A bliftering-plafter was alfo laid betwixt her fhoulders.

On the following day, (Sept. 14th), her fymptoms were very little mitigated. Her head was in the fame pofition, and the back and injured part of the neck was painful, though not much fwelled. She was reftlefs, efpecially with her hand, and would only lie on her right fide; for, by this time, fhe could turn herfelf in bed. Her jaw was equally as much locked, yet her pulfe was fuller, and more diftinct, though yet quick, and her breathing had become more natural. She had had no ftool, but had made water once, and previous to which, had given fome indication of her wants. The plafter had wrought well, and fhe was of a natural heat, unattended with any perfpiration. Her eyes were in the fame ftate, both with regard to the eye-ids, eye-balls, and immobility of the iris; and fhe had not yet attempted to fpeak. Ordered twelve drops of *tinct. opii* to be taken every hour, in a table fpoonful of the following mixture:

℞. Tinct. Valer. ℨiij. Tinct. Caftor ℨj. Aq. fontan ℥vij. M.

But previous to her taking this medicine, an emollient glyfter was ordered; but, through the obftinacy of her friends, was not adminiftered; and, contrary to my advice, loft nine ounces of blood, which feemed to fink the pulfe, without producing any effect on the jaw. To-day, however, fhe gave the firft indication of being fenfible; for, during the operation of bleeding, fhe kept her arm quite fteady, whereas before, it was almoft in a ftate of continued motion. She only took fix times of her medicine to-day, all of which made her ill, and gave her pain at her ftomach, of which fhe had no relief, till fhe threw up. The third, fifth, and fixth dofes were, however, only rejected.

The neck and left fide of the face were ordered to be rubbed with the following liniment, three or four times a day.

℞. Liniment. Ammon. ℥jfs. Tinct. Opii. ℨij. M.

She found great relief from this application, and fignified a wifh for a more frequent ufe of it.

15th. Her pulfe was fuller, and not fo quick, and during the night, fhe had frequent intervals of repofe; though fcarcely yet any alteration with regard to the jaw. She appeared, however, to be more fenfible, and frequently laid her hand on her forehead, and the back part of the neck.

The fmall degree of tenfion occafioned by the blow, was reduced, and the mufcles of the neck were more lax, though hitherto they exhibited no appearance of ftrong contraction. With refpect to the eyes, there was little variation; but fhe had nearly the power of fuftaining her head, which hitherto had been guided by the force of gravitation, and could bring it more more forwards, but not quite vertically. She was cool; had perspired none; took a little tea and chicken-broth, and sipped them through her teeth; had also made water once in the night, but yet had no stool. Ordered eight drops of tinct. opii to be given in a table spoonful of cold water every hour. This medicine she could only be persuaded to take once, as it again disagreed with her, but it was not rejected.

16th. She this day could distinguish colours, and even objects, notwithstanding her eyes were apparently in the same state. She slept moderately in the night, and made water plentifully; but even yet had no stool. Her pulse was becoming natural, and her appetite improving daily, but the jaw gave way very little. The musk mixture was ordered, but she refused taking it.

17th. Much the same as yesterday, touching the state of her jaw. Had slept moderately in the night, and had made water twice, and towards morning had a motion, by the aid of a suppository, composed of boiled treacle, with the addition of a little salt and butter. Her pulse was good, (at about 86), and she continued taking her liquid nourishment, but yet no solids, and signed for every thing she wanted; for yet she had not attempted to speak. She sat up an hour to-day, but with difficulty; could either bring her head forwards, or keep it steady; and she also walked three yards across the room. Ordered two-thirds of a grain of purified opium to be taken every three hours.

18th. She rested during the latter part of the night very well, and took five doses of her medicine, two of which she rejected almost immediately, and the rest stayed only whilst any liquid was taken into the stomach: When not thrown up, the opium never failed, as usual, to make her extremely ill, which she always signified by laying her hand on her stomach. Her pulse was approaching more and more to the natural standard. She had a loose stool to-day of a chocolate colour, and very offensive, and made water freely as usual. The jaw, though yet very stiff, was somewhat more free than yesterday. She can now hold her head vertically, but not long together, as it gives her great uneasiness. As to her eyes, there is no apparent alteration.

19th. No muscular contraction of the face, neck, or any other part appeared to exist; yet, notwithstanding, her jaw was considerably locked, but not so firmly but a moderate force would separate it one-tenth of an inch. In other respects, there was some variation, for she could now mutter some words, though very indistinctly. She made water twice to-day,

 but but had no ftool, and the ftate of her eyes were much as ufual. Ordered a tea-fpoonful of æther to be taken four times a day.

20th. Her jaw was fomewhat freer, as fhe could open it about one-feventh of an inch with her own hands. Her pulfe was become natural, and her fpirits good, and fhe walked more about the room. She ate minced meats now with a tolerably good appetite, and articulated fomewhat better, but was yet difficult to be underftood. She had one motion to-day, and made water twice, and faid her medicine did her much fervice, and was anxious to continue it. She had alfo fome little return of motion in the eye-balls, and they appeared lefs diftorted.

21ft. She articulated rather better, though yet very imperfectly; but her fpafm was daily giving way. She was cheerful and lively, and had regained fome ufe in the eye-lids and eye-balls. On clofing the eyes, the pupil became fo far depreffed, that the axis of vifion appeared parallel with the horizon; but on opening them, the pupil inftantly receded upwards. Some motion alfo was reftored to the iris, but too great a degree of light feemed offenfive to her, although fhe imprudently was amufing herfelf to-day with fome needle-work, but on reprefenting its impropriety, it was difcontinued. She complained fometimes of a little pain and ftiffnefs on the left fide of the jaw, at its articulation, but the bruifed parts exhibited no further marks of injury. She was without ftool to-day, but made water plentifully as ufual.

22d. Her jaw and fpeech were much the fame as yefterday; but in other refpects feemed rather worfe, occafioned probably by omitting her medicine. Her appetite was very indifferent, and her fpirits were dull and flat. She was ftill without ftool, and refufed all the means of procuring any. Ordered a grain of opium every three hours, in fubftance, along with a tea-fpoonful of the æther, in a table fpoonful of cold water, although it was with great difficulty fhe could be prevailed on to take the former medicine again.

23d. Slept well in the night, and had taken her medicine fix times, with good effects; not only in loofening the jaw, but in agreeing with the ftomach. Her fpirits were unufually good, and the appearance of her eyes more natural. She had alfo one motion, and made water as ufual, but her appetite was ftill indifferent.

24th. Difcontinued the opium yefterday, as fhe refufed taking it any longer, but the *æther* was perfifted in as before. The jaw continued giving way, and fhe could open it fo far, without affiftance, as to introduce her fore finger into her mouth. She fpoke alfo more intelligibly; I myfelf could comprehend feveral words, fuch as *milk*, *meat*, *no*, &c. and had now regained a tolerable ufe of her eyes and eye-lids, and could look in all directions; although yet, there feemed a ftrong tendency to look upwards, efpecially if fhe fixed her eyes ftedfaftly for a moment, either in a horizontal or oblique direction downwards. The iris had now regained its ufual power of contraction and dilatation. She had a natural ftool to-day, and made water well, but her appetite was yet indifferent. She gradually, however, recovered her fpeech and general health; but the jaw continued to give way very flowly, notwithftanding the ufe of æther a week longer; and even after the fpafm was apparently removed, fhe complained of a ftiffnefs a month or five weeks afterwards.

The urine throughout this diforder was plentiful, and natural in colour, and voided without pain or difficulty, a circumftance not frequent in *tetanus*. There was a ftrong tendency to coftivenefs, and to obviate which, moft of the neceffary means were denied; although, it muft be confeffed, that fhe experienced little or no inconvenience from it. The pulfe, foon after the accident, was 136, but daily became flower, and more diftinct, infomuch, that on the fifth day of her illnefs, they were at or below 90. The blood that was drawn was rather of a loofe texture, confidering the age and habit of the patient; and bleeding, under fimilar circumftances, I am perfuaded, will be inadvifeable.

As the locked-jaw is generally confidered as a fymptom of *tetanus*, and no other fign of this affection being here prefent, we may fairly refer the above cafe to fome other fpecies of fpafmodic affections. Here was no pain at the *fcrobiculus cordis*, in the thorax, or abdomen, nor pain or difficulty in fwallowing; and as to the *opisthotonos*, it may be confidered as rather proceeding from pain in the injured part of the neck, than from any mufcular contraction.

How far the circumftance of pregnancy, efpecially as being nearly the time of quickening, might have in conjunction with the accident, a power in occafioning the complaint; or whether the latter, independent of the former, was capable of producing it, are perhaps both equally doubtful and uncertain. One circumftance, which goes particularly towards characterizing this complaint a different fpecies of *trismus*, is, the very remedy, no lefs than a fpecific againft tetanus in general, not only difordering the ftomach, but bringing on a pain at the *fcrobiculus cordis*, which may be confidered as the pathognomonic fign of that affection.

Whence we may conclude, that the locked jaw is not neceffarily connected with, or dependent on *tetanus*; and that *opium*

is is not always a ſpecific in this complaint, for although here, when it ſtayed ſome time upon the ſtomach, its antiſpaſmodic virtues were diſcernible; yet from its unfriendly effects on that organ, a too frequent repetition of it would have been highly unwarrantable. So far from the opium being in this caſe a ſpecific, that it ſeemed to act only in a ſubordinate degree, requiring not only the æther to keep it on the ſtomach, but to prepare that organ for its reception; and even when it was retained ſome time before it was rejected, its antiſpaſmodic effects were not what might have been expected. One particular phenomenon manifeſted itſelf with regard to the eye; for, notwithſtanding the diſtortion of the eye-ball, and the preternatural dilatation and immobility of the *iris*, the patient could diſcern not only colours but even minute objects. The day after bleeding, ſome vivid colour (I believe blue) was accidentally expoſed to her ſight; and her friends were ſenſible of her ſeeing it. This circumſtance gave riſe to a few experiments, the reſult of which proved, that ſhe could ſee or diſcern objects when placed at a certain diſtance. For inſtance, a pen-knife was placed at two inches diſtance, and ſhe ſignified what it was. A half-penny and a pin were alſo placed at certain diſtances, the latter at about one inch, which ſeemed to be the proper *focus*. Theſe ſhe alſo diſcovered; but the pen-knife ſhe could not ſee at ſix inches diſtance. When theſe experiments were made, the eyes were exactly in the ſame ſituation as deſcribed in the narrative; namely, about two-fifths cloſed, and exhibiting little more than the diſk of the cornea, if it may be ſo called, through the eye-laſſes: At this time, on opening the eye-lids, ſo as to expoſe the cornea to the effects of the light, there appeared a ſlight oſcilation of the eye-ball upwards; but on cloſing the eye-lids, it reſumed its former ſituation.

This ſtate of the eyes, with the nature of viſion, tend much to favour the theory of *Dr. Hosack* of *New York*, who maintains, that the *recti* muſcles of the eye contribute much in lengthening or ſhortening the axis of viſion, by enlarging or diminiſhing the refracting medium; and that in proportion as the axis becomes elongated, the eye is rendered leſs capable of ſeeing objects at a diſtance, as was the caſe here.

*Mr. Young*, in the *Philoſophical Tranſactions*, ſuppoſes the ſole uſe of the retina and optic nerve is, to convey ſenſation to the brain; and this caſe perhaps alſo favours his conjecture, ſince the patient could ſee very well, when the object was applied at a proper diſtance from the eye, notwithſtanding the iris exhibited no ſenſibility when expoſed to the light.*

For

* Her eyes would have been expoſed to a ſtrong candle-light, had not the obſtinacy of her friends prevented me.

For if the fenfitive power of the iris depends on any connection with the *retina*, why fhould the function of the former be fufpended, whilft the office of the latter remained unimpaired? There feems little doubt of the nerves which fupply the mufcles and coats of the eye being affected; and equally the fame certainty of the retina being unaffected; and confequently in this diftorted and preternatural ftate of the parts, no apparent connection may be thought to exift betwixt the one and the other; yet this inference is far from being conclufive, when applied to the natural ftate of the eye, as the phenomena produced by the deranged ftate of that organ feem incompetent to folve any of the difficulties relating to vifion in general. The circumftance of the retina retaining its function whilft the power of the iris was apparently deftroyed, may probably, on a due confideration of the cafe, be accounted for, more efpecially if we take into the account the alteration effected both in the appendages, form, and contents of the eye. Here we fuppofe the *elevator* mufcle to be ftrongly contracted, which would draw up the eye-ball along with it, thereby making only a fmall preffure on its contents; whereas, the counteracting force of the *depreffor* would prefs fo powerfully on the under part of this organ, as neceffarily to pufh up its contents, deftroy its globofity, and confequently to diminifh its cavity. Hence the cryftalline humour is pufhed forward through the anterior chamber, which would not only enlarge the pupil, but prevent its contraction. That the pupil becomes enlarged in proportion as the axis of vifion is elongated, it feems evident from a *fpeculum* preffing ftrongly on the globe of the eye, and which increafes the dilatation thereof in a correfponding degree; and befides, during this enlargement of the pupil, whilft this inftrument is applied, little or no motion is difcernible in the iris, when the eye is expofed to alternate light and darknefs. From the fame reafons alfo that the pupil is enlarged as the axis of vifion is lengthened, the former becomes leffened as the latter is fhortened. This is particularly exemplified in youth and old age.

In the month of July following, I again faw this young woman, who complained of weaknefs and watering of her eyes, efpecially on looking downwards, and could not bear to look ftedfaftly at any object. She complained alfo of pain in the head, which it appeared fhe had been fubject to, efpecially in the fummer feafon, and which fhe defcribed as now fhooting from the fore to the back part of the head, and downwards to the original feat of the injury. She ftill was pofitive fhe could diftinguifh any object, or colour, after fhe became fenfible, if held at a proper diftance from her eyes. She made water as before before related, the night after the accident, and figned for the neceffary affiftance, as fhe always did afterwards. Notwithftanding this circumftance, fhe never recollected any thing from the time of the accident (being Thurfday), till the Sunday following; the clock ftriking one, being the firft thing fhe could remember. She was confident the *opium* always made her ill, and caufed pain at her ftomach, efpecially when taken without the æther, which was relieved only by vomiting; and when taken in conjunction with this medicine, its unpleafant effects were nearly obviated; when the æther was, however, taken by itfelf, fhe faid it did her more fervice; and, according to her own expreffion, always revived her. The eyes appeared diminifhed in fize, and the iris to contract very powerfully. The pupil alfo of the right eye, when in a ftate of contraction, affumed an oval form.

In juftice to Mr. Pearfon, (by whofe hand we were favoured with Mr. Bradley's Cafes in Surgery) it may be proper to fay, that the teaching of the medical practice requifite in Surgery has been his principal object during a feries of eighteen years, as the text book of his Lectures evinces. He is, we believe, the firft perfon in this kingdom who undertook to reduce the principles of Surgery into a fcientific form, and to explain at great length, all the moft important chirurgical difeafes, and the mode of treating them. Each of his Courfes contains a much greater number of Lectures on Surgery, (although he includes all the operations) than any Courfe ever delivered in London. EDIT.

